# Do Antioxidant Vitamins Prevent Exercise-Induced Muscle Damage? A Systematic Review

**DOI:** 10.3390/antiox9050372

**Published:** 2020-04-29

**Authors:** María Martinez-Ferran, Fabian Sanchis-Gomar, Carl J. Lavie, Giuseppe Lippi, Helios Pareja-Galeano

**Affiliations:** 1 Faculty of Sports Sciences and Physiotherapy, Universidad Europea de Madrid, 28670 Madrid, Spain; maria.martinez.nutricion@gmail.com; 2 Department of Physiology, Faculty of Medicine, University of Valencia, 46003 Valencia, Spain; fabian.sanchis@uv.es; 3 INCLIVA Biomedical Research Institute, 46003 Valencia, Spain; 4 Division of Cardiovascular Medicine, Stanford University School of Medicine, Stanford, CA 94305, USA; 5 John Ochsner Heart and Vascular Institute, New Orleans, LA 70121, USA; clavie@ochsner.org; 6 Ochsner Clinical School, The University of Queensland School of Medicine, New Orleans, LA 70121, USA; 7 Section of Clinical Biochemistry, University of Verona, 37129 Verona, Italy; giuseppe.lippi@univr.it

**Keywords:** antioxidant vitamins, vitamin C, vitamin E, muscle damage

## Abstract

Free radicals produced during exercise play a role in modulating cell signaling pathways. High doses of antioxidants may hamper adaptations to exercise training. However, their benefits are unclear. This review aims to examine whether vitamin C (VitC) and/or vitamin E (VitE) supplementation (SUP) prevents exercise-induced muscle damage. The PubMed, Web of Science, Medline, CINAHL, and SPORTDiscus databases were searched, and 21 articles were included. Four studies examined the effects of acute VitC SUP given pre-exercise: in one study, lower CK levels post-exercise was observed; in three, no difference was recorded. In one study, acute VitE SUP reduced CK activity 1 h post-exercise in conditions of hypoxia. In three studies, chronic VitE SUP did not reduce CK activity after an exercise session. Chronic VitE SUP did not reduce creatine kinase (CK) concentrations after three strength training sessions, but it was effective after 6 days of endurance training in another study. Chronic SUP with VitC + E reduced CK activity post-exercise in two studies, but there was no such effect in four studies. Finally, three studies described the effects of chronic VitC + E SUP and long-term exercise, reporting dissimilar results. To conclude, although there is some evidence of a protective effect of VitC and/or VitE against exercise-induced muscle damage, the available data are not conclusive.

## 1. Introduction

In the human body, the antioxidant system consists of antioxidant enzymes and non-enzyme antioxidants [1]. The latter are classified as lipid soluble, when present in membranes and lipoproteins, or water soluble, when found in extracellular and intracellular fluids [1,2]. Oxidative stress is the consequence of an imbalance between reactive oxygen species (ROS) production and antioxidant defenses [3]. This stress can lead to damage to cell components and may have detrimental effects in both physiological conditions, such as during physical exercise, and disease conditions [3,4].

While it has been well established that regular physical activity has notable health benefits [5], repeated skeletal muscle contractions generate free radicals and, when prolonged and intense, exercise can cause oxidative damage to cells, particularly in untrained persons [4,6]. The main endogenous sources of ROS in skeletal muscle are mitochondria and the enzymes nicotinamide adenine dinucleotide phosphate (NADPH) oxidase and xanthine oxidase (XO). This last enzyme is thought to contribute to exercise-induced muscle damage, initiating damage to membrane lipids [7]. In addition, free radicals generated by neutrophils seems to produce muscle damage [8]. Lipid peroxidation increases membrane permeability and induces cellular damage [9]. Accordingly, it has been proposed that the intake of antioxidants could prevent exercise-induced muscle damage.

Today, there is evidence that free radicals produced during exercise play a role in modulating cell signaling pathways and many redox-sensitive transcription factors [6]. High doses of antioxidants may hamper certain adaptations to exercise training [10,11,12]. However, despite the unclear benefits of antioxidant supplements, consumption of vitamin C (VitC) and vitamin E (VitE) has recently increased [4]. VitC or ascorbic acid, a water-soluble antioxidant, is the first line of antioxidant defense in the human body [13]. This vitamin has multiple antioxidant actions, as it is able to react with several free radicals and ROS [2,14]. VitE is a lipid-soluble antioxidant that refers to eight structural isomers of tocopherol and tocotrienol [6]. The biologically and chemically most active form of VitE, alpha-tocopherol, is the most abundant lipid-soluble antioxidant in humans and protects against lipid peroxidation [2,14].

The extensive use of antioxidant vitamins associated with physical exercise makes it necessary to determine their effects on health and the physiological adaptations pursued by training. VitC and VitE are the most widely used antioxidant vitamins among exercisers. Antioxidants in physical exercise and exercise training, particularly VitC and VitE, remain a hot topic in sports nutrition, exercise physiology and biology [15]. Antioxidants have received attention as a strategy for preventing or minimizing the negative effects of reactive oxygen and nitrogen species (RONS) generated during and after exercise training. VitC and VitE SUP has become a common practice among exercisers in order to reduce oxidative stress, accelerate recovery and enhance performance [16]. However, its requirements and effects have not been established sufficiently. Therefore, there is a need to determine the real effects of VitC and/or VitE in exercise training as well as obtain useful conclusions and practical implications based on recent evidences.

In this systematic review, we examine whether short- or long-term VitC and/or VitE SUP is capable of preventing acute or chronic exercise-induced muscle damage in athletes, and in physically active or inactive individuals. The impacts of vitamin intake were assessed through the muscle damage indicator creatine kinase (CK), and the lipid peroxidation, or oxidative stress, markers malondialdehyde (MDA) and thiobarbituric acid-reactive substances (TBARS).

## 2. Materials and Methods 

### 2.1. Eligibility Criteria

The PICOS model eligibility criteria considered for study inclusion were population, intervention, comparators, outcome and study design [17]. The study populations considered were healthy male and female subjects of any age who were sedentary, physically active, or professional athletes. The interventions considered were chronic or acute SUP with a defined dose of VitC and/or VitE, and subjects must have undertaken any form of acute or chronic standardized physical exercise or sport after acute SUP or a SUP period. Comparators were effects observed for placebo versus VitC and/or VitE. Outcomes reported were muscle damage, measured as CK levels. When available, other muscle damage biomarkers, such as MDA, TBARS, myoglobin (Mb), and lactate dehydrogenase (LDH), were also reported. Finally, the design of the studies should be single- or double-blind and randomized. 

The following papers were excluded: those describing observational studies (cohort, case-control, ecological, case reports, and case series) or animal studies; studies conducted in injured or ill participants; studies in which VitC and/or VitE were administered with other supplements; articles with no full-text available, opinion pieces, review articles, commentaries and editorials.

### 2.2. Literature Search

This systematic review was conducted and reported according to Preferred Reporting Items for Systematic reviews and Meta-Analyses (PRISMA) guidelines [18]. To identify all relevant articles, the PubMed, Web of Science, Medline, CINAHL, and SPORTDiscus databases were searched using the terms [(“vitamin C” OR “vitamin E”) AND (“muscle damage”) AND supplementation]. All original full-text articles in English or Spanish published before 31 August 2019 were considered. 

### 2.3. Study Selection

A two-stage search strategy was employed after duplicates were removed by a reviewer. In the initial stage, titles and abstracts were screened to exclude irrelevant articles according to eligibility criteria. At this stage articles of questionable suitability were included. In the second stage, full texts of the investigations identified in the first stage were read to determine whether they met the inclusion criteria. Reference sections of relevant articles were also examined via the snowball strategy.

### 2.4. Data Extraction

The following information was extracted from the selected articles: study source (authors and year of publication), participant characteristics (level of activity or sports discipline, number of participants, age, sex and gender), intervention protocol (SUP type and dose, period of SUP, type of sport/exercise), main outcome (CK), and other outcomes (changes in other muscle damage indicators, i.e., LDH, Mb, plasma VitC and VitE, lipid peroxidation measured as MDA or TBARS/TBA, performance, and muscle soreness).

### 2.5. Quality Assessment and Risk of Bias

The quality of each investigation was assessed following the Cochrane Collaboration Guidelines [19]. The Cochrane Risk of Bias tool for randomized clinical trials is based on seven domains: sequence generation and allocation concealment (selection bias), blinding of participants and personnel (performance bias), blinding of outcome assessment (detection bias), incomplete outcome data (attrition bias), selective reporting (reporting bias) and other sources of bias (other bias). Risk of bias was categorized as low, high or unclear.

### 2.6. Presentation of Results

Findings were individually assessed and described based on the information provided by each of the reports included.

## 3. Results

### 3.1. Study Selection

**Figure 1:**
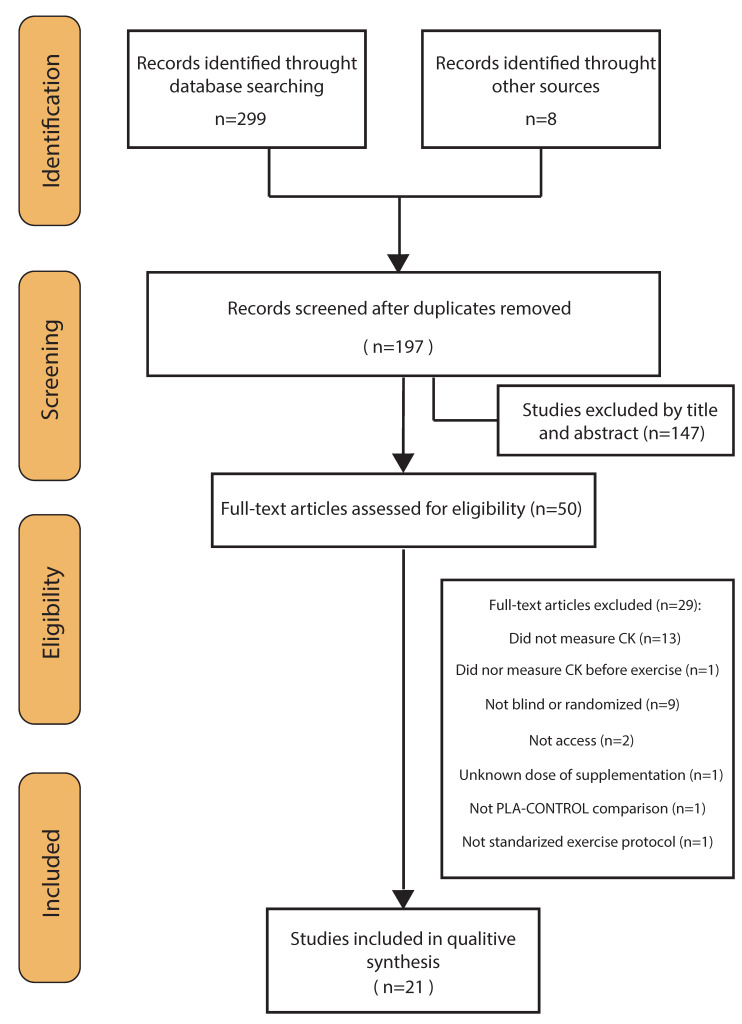
Flow diagram of literature search according to the PRISMA statement.

Through the database searches, 299 records were identified. Eight additional studies could be identified through other sources (Figure 1). Of these records, 110 duplicates were removed, and 147 articles excluded after screening the title and abstract for eligibility. This left 50 studies, which were assessed for eligibility. Of these 50 studies, another 29 were removed according to the inclusion/exclusion criteria and two because full texts were unavailable. The studies finally included were 21 randomized placebo-controlled trials, 19 double-blind and 2 single-blind trials [20,21]—four of which had a crossover design [20,22,23,24]. See Figure 1 for more details.

### 3.2. Characteristics of the Studies

In total, there were 369 participants in the 21 studies (338 men and 31 women). Most studies had <25 participants, and only two assessed the effects of antioxidant vitamins in a larger group of subjects (*n* = 32 and *n* = 64). Healthy untrained individuals were selected in five studies and healthy physically active participants in another five. Participants of the remaining studies were classified as athletes as they trained for a specific sport: running (*n* = 4), soccer (*n* = 2), basketball (*n* = 1), taekwondo (*n* = 1), weightlifting (*n* = 1), resistance training (*n* = 1) and cycling (*n* = 1). Most of the studies included participants aged between 18 and 30 years. However, in three studies, participants were under 18 years, in two between 33 and 40 years, and one study included two groups aged 22 to 29 years and 55 to 74 years.

Acute doses of antioxidant vitamins were administered 1 to 3 h before testing in five of the studies. In the rest, daily vitamin SUP was taken from 4 days to up to 5 months. The SUP tested was VitC in four studies, VitE in eight and both in nine. Doses given were 200 to 2000 mg for VitC, and 250 to 1400 mg for VitE. Sources of VitE were dl-α-tocopheryl acetate, α-tocopherol, and RRR-d-α-tocopherol succinate.

### 3.3. Quality Assessment and Risk of Bias

In all studies, random sequence generation was categorized as low bias risk and allocation concealment as unclear in all studies but one. In terms of performance and detection bias, 19 (double-blind) studies were characterized as low risk and two (single-blind) studies as high risk.

**Figure 2:**
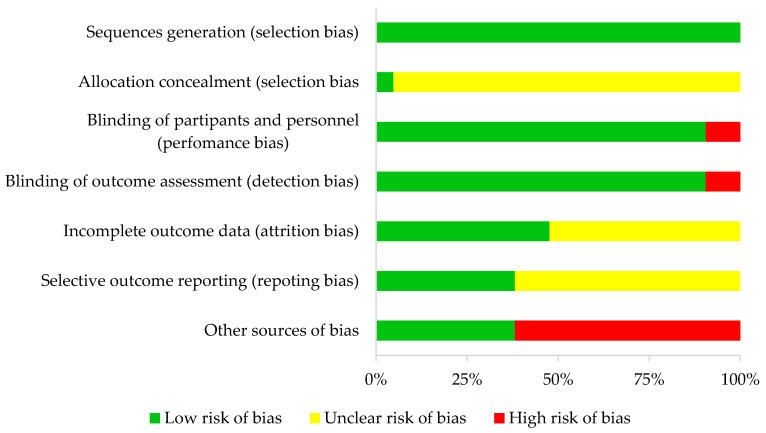
Risk of bias summary.

For attrition bias, ten studies were categorized as low risk and eleven as unclear risk. For reporting bias, eight articles were considered low risk and thirteen unclear risk. Finally, eight studies were described as having an overall high risk of bias and thirteen a low risk of bias. This information is detailed in Table 1 and Figure 2.

### 3.4. Results of Individual Studies

The studies included in this review were clustered by SUP and exercise protocol: five studies examined the effect of acute SUP in a single exercise session (Table 2), thirteen examined the effect of chronic SUP in an acute exercise session (*n* = 11) or several exercise sessions per week (*n* = 2) (Table 3), and three studies examined the effect of chronic SUP over a long-term exercise period (Table 4). See Supplementary Materials for additional data on the studies.

#### 3.4.1. Acute Supplementation

Nakhostin-Roohi et al. [29] and Bohlooli et al. [28] investigated the effect of VitC SUP (1000 mg and 500 mg) given 2 h before a 30 min running session at 75%VO_2max_ in untrained men. Both studies found no significant differences in CK concentration between groups. Nevertheless, CK levels increased significantly immediately and 2 h after exercise in both groups, but only remained elevated 24 h after exercise in the placebo group. In both studies, the lipid peroxidation indicator MDA was elevated 2 h post-exercise only in the placebo group, but without significant differences compared to the SUP group.

Nie and Lin [32] observed that the intake of 800 mg of VitC 3 h before and 21 h after performing eccentric exercise led to lower CK concentrations 24 and 48 h afterwards in junior basketball players compared with placebo. In both groups, MDA was elevated post-exercise and remained so 24 h later. In contrast, no effect of VitC SUP on CK was reported by Thompson et al. [24], who administered a 1000 mg dose to physically active men 2 h before the Loughborough Intermittent Shuttle Test (LIST). In both groups, MDA increased significantly immediately post-exercise. 

Finally, Santos et al. [23] noted that SUP with 250 mg of VitE 1 h before 60 min of exercise under conditions of hypoxia led to reduced CK levels 1 h after exercise in physically active men and levels were also reduced in comparison to exercise performed under normoxia. Compared to conditions of hypoxia and intake of placebo, CK-MB and lactate dehydrogenase (LDH) were also reduced 1 h after exercise.

#### 3.4.2. Chronic Supplementation

##### Acute Exercise Protocol

In weight-trained men taking 1200 IU of VitE per day for 2 weeks, McBride et al. [21] observed significantly reduced CK activity 24 h after high-intensity resistance training compared with placebo, while there was no significant difference in MDA concentration between groups at any measured time point (i.e., immediately post-, 6 h post-, 24 h post-, and 48 h post-exercise).

Beaton et al. [34] also examined the effects of SUP with 1200 IU of VitE over 30 days in sedentary men after a bout of 240 maximal isokinetic eccentric muscle contractions. Although CK activity increased at the different time points established (i.e., immediately post- and 48 h post-exercise), the authors reported no difference in CK activity between groups (see Table 2). 

Niess et al. [22] provided non-physically active subjects with a dose of VitE (500 IU/d) for 8 days before they completed a combined exhaustive treadmill protocol. Despite no differences between groups, they observed that CK remained elevated in the placebo but not in the SUP group at 48 h post-exercise. 

Cannon et al. [40] assessed the effects of VitE SUP (800 IU/d over 48 days) in younger and older men after 45 min of downhill running on a treadmill. These authors found that in the older SUP group, CK was significantly higher before exercise and 2 days afterwards than in the older placebo group. Supplementation tended to reduce CK in young subjects, but not significantly. Compared to the younger placebo group, older men given placebo had significantly lower levels of CK. There was no increased production of MDA at any time point and no difference between groups. 

In studies by Chou et al. [26] and Rokitzki et al. [37], significantly lower CK concentrations were observed after exercise in the SUP group than in placebo group. Chou et al. [26] administered VitC (2000 mg/d) and VitE (1400 IU/d) for 4 days before a simulated competition to competitive Olympic Taekwondo athletes. A lower area under the curve for CK and Mb, and lower Mb were detected in the SUP group during the competition. However, there were no significant differences between groups in CK concentrations at any time point. Rokitzki et al. [37] supplemented runners with VitC (400 mg/d) and VitE (400 IU/d) for 4.5 weeks before a marathon and found that 24 h after exercise, CK was lower in the SUP group. Notwithstanding, these authors also observed that CK was significantly higher in the SUP group before SUP. No significant differences emerged between the SUP and placebo groups in TBARS, whose levels fell immediately after the race and then increased 24 h later. 

Mastaloudis et al. [31], Dawson et al. [20] and Petersen et al. [35] investigated the effects of different doses of VitC plus VitE on CK concentrations in runners (500–1000 mg/d of VitC and 300–1000 IU/d of VitE) taken for 6 weeks, 4 weeks, and 2 weeks before a 50 km ultramarathon, a marathon and a 1.5 h downhill exercise, respectively. In none of these studies were differences in CK levels detected between SUP and placebo groups. In addition, Mastaloudis et al. [31] and Dawson et al. [20] observed no differences in LDH or Mb, while Dawson et al. [20] noted no differences in MDA. 

De Oliveira et al. [25] examined the effects of SUP with 500 mg/d of VitC plus 400 UI/d of VitE 7 days before and 7 days after an exercise-induced oxidative stress protocol in soccer players. Although no significant differences in CK were found between groups, its activity was reduced by 34% in the SUP group. These authors also found that MDA and lipid hydroxyperoxide were significantly elevated 24 and 48 h after exercise compared with placebo group.

Jakeman and Maxwell [39] noted no differences in CK measured in active men and women after 60 min of box-stepping following 21 days of VitC (400 mg) or VitE (400 IU) intake. In the physically active men, the supplements were also given 3 weeks before and during three whole-body resistance exercise sessions separated by 3 days. A higher CK area under the curve was found in the VitE group while the placebo and SUP groups did not differ in plasma MDA levels, which were elevated on days 7 and 8 post-exercise.

Avery et al. [33] and Itoh et al. [36] examined the effects of SUP with 1200 IU/d of VitE after different sessions of exercise over a week. In the first study, the vitamin was given to active men 3 weeks prior to and during 3 whole-body resistance exercise sessions separated by 3 days. A higher CK area under the curve was found in the VitE group. No significant difference between groups was found in plasma MDA, which increased on days 7 and 8 post-exercise. In the second study, after supplementing trained male runners for 4 weeks before and during a 6 day running training session, it was found that SUP led to significantly reduced concentrations of both CK and LDH 24 h after exercise. Further, in the placebo group, lipid peroxidation measured as TBA was higher the day before the trial, on the next day and 3 weeks later.

##### Chronic Exercise

Two of the three investigations designed to examine the effects of VitC and/or VitE over a long-term period of exercise detected reductions in CK activity. Zoppi et al. [30] found that the intake of 1000 mg/d of VitC plus 800 IU/d of VitE over 3 months of a pre-competition season in professional soccer players reduced CK at the end of the season compared with placebo, but not at mid-season. Levels of lipid peroxidation measured as TBARS were also blunted at the end of the season compared to those recorded in placebo group. Rokitzki et al. [38] reported that VitE (330 IU/d) taken over 5 months of an aerobic exercise training program in professional racing cyclists led to reduced CK and MDA at the end of the program compared to placebo, both before and after performing an acute incremental test until exhaustion. No significant changes were seen for LDH. In contrast, Mohammed et al. [27] reported no effects of VitC (500 mg/d) plus VitE (400 IU/d) taken over 6 weeks on levels of CK or LDH in competitive weightlifters carrying out their routine training 3 days per week.

## 4. Discussion

The results of seven of the twenty-one studies reviewed suggest that VitC and/or VitE SUP may be associated with reduced CK release into the bloodstream in response to exercise. This finding could point to a beneficial effect of antioxidant SUP in mitigating the muscle damage induced by exercise through muscle membrane disruption. Three further studies also concluded that vitamin SUP could expedite the return to baseline levels of CK after exercise, although differences between the placebo and SUP groups did not reach statistical significance. Unlike these findings, eleven articles reported no CK reducing effect of SUP with these vitamins, although reductions were observed in two studies, albeit not significant reductions. 

The studies reviewed were heterogeneous in terms of SUP protocols (acute or chronic SUP with different doses of VitC or/and VitE), participants (professional athletes, recreational athletes, and physically inactive subjects) and exercise protocols (acute or chronic exercise; different sports or types of exercise). This determines a need for further work with standardized protocols so that valid conclusions can be drawn. 

VitC SUP, alone (*n* = 4) or in combination with VitE (*n* = 9), was tested in 13 studies in which doses ranged from 200 to 2000 mg. Meanwhile, VitE, alone (*n* = 8) or in combination with VitC (*n* = 9), was tested in 17 studies in which doses ranged from 250 to 1400 mg from dl-α-tocopheryl acetate, α-tocopherol, and RRR-d-α-tocopherol succinate sources. All antioxidant vitamin doses used in the studies included were above the Dietary Reference Intake (DRI) according to the European Food Safety Authority (EFSA) [41,42] and then could be considered a “pharmacological dose.”

Lipid peroxidation is thought to lead to sarcolemma disruption [43]. Thus, because of their antioxidizing properties, VitC and/or VitE could mitigate lipid peroxidation. Regrettably, lipid peroxidation measurements were not performed in several studies included in this review [22,23,26,31,34,35,39]. Further, some authors did not detect differences in CK or lipid peroxidation markers between placebo and SUP [20,24,27,33,40]. Notwithstanding, Oliveira et al. [25] did detect higher MDA concentrations in their placebo group 24 h after exercise, while CK activity was similar between groups. In contrast, Rokitzki et al. [38] and Zoppi et al. [30] observed that markers of lipid peroxidation (TBARS or MDA) were blunted at the same time points as CK. Bohlooli et al. [28] found that the total antioxidant capacity decreased in the placebo group 2 and 24 h after exercise compared with the VitC SUP group. However, the placebo group but not the VitC group increased MDA levels after exercise. Nakhostin-Roohi et al. [29] observed that MDA levels increased 2 h after exercise only in the placebo group compared with the VitC SUP group while the total antioxidant capacity decreased only in the placebo group when compared with baseline. Interestingly, the study found that the Vit C SUP group increased lymphocyte counts immediately after exercise compared with baseline while serum cortisol declined after VitC SUP compared with baseline remaining low at 2 and 24 h after exercise. Contrary, Nieman et al. [44] found that VitC SUP does not serve as a countermeasure to oxidative changes, measured through the blood levels of lipid hydroperoxide and F2-isoprostane, during or after a competitive ultramarathon race. McBride et al. [21] showed that MDA increased in both the placebo and VitE SUP groups when administered chronically. However, the lipid peroxidation dynamics were different between both groups. The placebo group showed increased MDA levels at 6 and 24 h post-exercise while the MDA levels in the VitE SUP group only increased immediately after exercise. Itoh et al. [36] observed that without the exercise stimulus, VitE SUP reduced the TBA levels when compared with placebo. Contrary, Nieman et al. [45] indicated that lipid peroxidation, measured as plasma F2-isoprostanes, could be increased after VitE SUP when compared with placebo. It seems that limiting lipid peroxidation could be one of the mechanisms of action of antioxidant vitamins in reducing muscle damage.

One of the many theories proposed to explain the mechanisms underlying delayed onset muscle damage (DOMS) is the increased production of free radicals and ROS [46]. This explains the popular belief that ingestion of antioxidant vitamins will reduce muscle soreness after exercise [47,48]. Some studies reviewed here assessed the impacts of antioxidant vitamin intake on perceived muscle soreness [21,24,25,32,33], although no such impacts were detected. Of these studies, only that published by Nie and Lin [32] detected an effect of SUP on muscle damage. Accordingly, these authors proposed that the inflammatory response involved in muscle soreness is independent of any change produced in CK. This rationale is in line with results of a recent Cochrane review, which found moderate to low evidence supporting the use of "antioxidant" SUP for preventing or lowering DOMS [48]. Additionally, Close et al. [49] demonstrated that VitC SUP did not affect DOMS after downhill running but may inhibit the recovery of muscle function.

Another effect attributed to antioxidant vitamins is enhanced performance, although consistent evidence of this is lacking [16,50,51]. Some studies reviewed here included performance measurements. When subjects took antioxidant vitamin supplements over a chronic period and were then tested in an acute exercise session, no differences between SUP and placebo groups were found in different performance indicators: isometric torque or concentric torque [34]; maximal isometric strength in squat and bench press [33]; maximal voluntary contraction (MVA), eccentric hamstring torque and concentric quadriceps power [31]; vertical jump, agility, sprint time or fatigue index [25]; and aerobic work capacity [36]. Interestingly, a recently published systematic review and meta-analysis by Dutra et al. [52] concludes that VitC and VitE SUP has no effect on muscle force production in chronic strength training and could even attenuate specific adaptions such as hypertrophy.

In addition to the notion that antioxidant SUP does not improve exercise performance, there is also growing evidence to suggest that chronic antioxidant supplementation may interfere with normal skeletal muscle adaption process. In effect, ROS produced during exercise regulates many physiological processes, acting as signals to modulate the adaption of muscles to exercise [10,11,12,15,47,53]. Gomez-Cabrera et al. [53] found that VitC SUP hampered training-induced adaptations in endurance performance both in murine and human models. Their results in rats demonstrated that the ROS produced during exercise activates the expression of the antioxidant enzymes manganese-dependent superoxide dismutase (MnSOD) and glutathione peroxidase (GPx) in skeletal muscle. However, when ROS was blunted with VitC SUP, the endogenous antioxidant defense was prevented. These authors also found that the exercise-associated ROS production up-regulated mitochondrial biogenesis, measured by peroxisome proliferator-activated receptor-gamma coactivator-1alpha (PGC-1α), nuclear respiratory factor 1 (NRF-1) and mitochondrial transcription factor A (TFAM), and mitochondrial content evaluated by the cytochrome C content. However, all these biomarkers of mitochondrial biogenesis and content were blunted when VitC SUP was administered [15,53]. Similar conclusions have been obtained through the inhibition of a free radical-generating enzyme (xanthine oxidase) by allopurinol, which severely attenuates exercise activation of the mitochondrial biogenesis pathway in skeletal muscle [54,55]. However, allopurinol has been shown to reduce several biomarkers of exercise-induced muscle damage in different sports specialties such as soccer [56,57], cycling [58] or marathon [59], suggesting potential therapeutic approaches [60]. Hence, although some studies included in this review showed that chronic antioxidant SUP could reduce muscle damage in response to exercise, the evidence available so far also suggests that it may even impair muscle adaptations [11,12,15,53].

## 5. Limitations of the Current Evidence

As the main limitation of the studies reviewed, with the exception of two studies [27,38] (*n* = 30 and *n* = 32, respectively), most had <25 participants, so statistical power was low. Some studies did not mention whether participants avoided exercise before or during the SUP period, which could have influenced the level of muscle damage [23,28,33,39]. Another critical factor is that in some studies, the participants were familiar with the exercise protocol, and in others, they were not, or this was not specified. This might have affected the results, as a single bout of unfamiliar exercise protects against subsequent damage in response to the same type of exercise [61]. The study by Beaton et al. [34] had the limitation that CK levels were analyzed 72 h and 7 days after exercise, despite reports that CK peaks at 24 h post-exercise [62]. In another study [40], a biopsy was taken from the younger participant group immediately before exercise and 5 days after exercise. This could explain why lower CK levels were detected in the older than younger placebo group subjects. In some studies conducted in sedentary participants, a maximum oxygen consumption (VO_2max_) test was performed one week [23], at least two weeks before [28,40] or several months before [40] before the trial. However, participants were not familiar with the exercise protocol. Neither did the subjects in the study by Niess et al. [22] have experience in the exercise test before the trial. The subjects recruited by Beaton et al. [34] and Avery et al. [33] were not resistance-trained athletes and were not accustomed to eccentric exercise tests. In the study by Nie and Lin [32], although subjects played in the same team, it was not specified whether they were familiarized with the eccentric exercise. In most investigations, participants were instructed to avoid the use of other supplements. However, this was not mentioned in some of the papers [21,23,32,37]. In some of the studies, plasma levels of VitC or/and VitE were not determined [20,21,23,26,27,30,33], so it is not known whether baseline levels differed in the placebo and SUP groups and this could have influenced the results.

Nutrition plays an essential role in performance and recovery from physical exercise [63]. Furthermore, some diet components can help recovery from exercise-induced muscle damage, such as the intake of protein and its timing [64]. As a result, not controlling participants’ diet and not avoiding differences between groups could have influenced the results of the studies reviewed here. Antioxidant status, evaluated through dietary intake and/or plasma levels of VitC or VitE metabolites, has been analyzed in eleven of the twenty-one studies included in this systematic review. Plasma levels of VitE (alpha-tocopherol) do not reflect dietary intake or body reserves, since only 1% of body tocopherol is circulating in the bloodstream [65]. In five studies of the included, the dietary intake of VitC and VitE was assessed through questionnaires considering one or a few days. However, different methods were used. None of the studies detected significant differences between groups in dietary intake of VitE or VitC. Nonetheless, in some of them, intakes were below the recommended dietary allowance based on the Institute of Medicine recommendations [66]. In most, dietary intake of VitE was below recommendations [24,25,30,31] and only Nie and Lin [32] reported an adequate intake. It has been suggested that VitE deficiency could impair muscular endurance and alter muscle contractile properties [67]. It should also be considered that dietary assessments are typically poorly specific and scarcely sensitive [65]. The dietary intake of VitC was below the recommended level in one article [30] and adequate in three of them [24,25,31,32]. Accordingly, since only five of the twenty-one studies included evaluated the adequate or inadequate intake of VitC and/or VitE, we cannot confirm a relationship between the previous intake of antioxidant vitamins on the results of muscle damage analyzed.

Finally, in the three studies focusing on the impacts of SUP during chronic exercise [27,30,38], participants had the same training loads. However, in the study carried out by Mohammed et al. [27], participants were asked to perform the training protocol individually while participants trained together in others. Other differences are that in the studies of Zoppi et al. [30] and Rokitzki et al. [38], participants undertook an initial preparation period, while [27] participants were trained before starting the exercise protocol in the study of Mohammed et al. In this study, program intensity was set to 70%, 80%, 90%, and 100%, respectively, during the first 4 weeks and then cut back to 70% and 80% for the last 2 weeks of intervention. As muscle damage was only assessed at the beginning and at the end of SUP, it would be interesting to know the level of damage produced at the 100% training intensity.

## 6. Conclusions

The studies reviewed provide some data, although relatively weak, indicating a protective effect of antioxidant vitamins against exercise-induced muscle damage. However, this evidence is not conclusive, and not all articles reported a clear benefit from VitC and VitE SUP. Results also suggest that the protective role of antioxidant SUP could be more related to protection against exercise-induced lipid peroxidation than to muscle damage as measured by plasma CK levels. On the basis of the current evidence reported by numerous studies with different functional measurements, acute or chronic antioxidant vitamin SUP does not seem to benefit physical performance.

Furthermore, while antioxidant vitamins might reduce muscle damage, it seems that chronic SUP could impair muscle adaptations to training [12,15,47]. This means that VitC and VitE supplements should probably not be given to athletes during training, when muscle adaptations are pursued. In contrast, acute SUP with antioxidant vitamins could lessen muscle damage and thus improve recovery while training or during consecutive competitions. Additionally, in sedentary individuals, while antioxidant SUP could mitigate the muscle damage induced by an acute session of exercise, regular exercise will offer the most significant benefits, as physical exercise seems to have an antioxidant effect per se [68].

Overall, it appears to be an invalid assumption to prescribe athletes VitC and VitE as antioxidant supplements to avoid oxidative stress and, subsequently, performance. Unfortunately, very few well-conducted studies have been performed to evaluate the efficacy of VitC and VitE supplements, making recommendations in this regard very difficult. From our point of view, a balanced diet is more than enough to maintain an appropriate antioxidant status.

## Figures and Tables

**Table 1:** Risk of bias graph.

Study	Sequences Generation (Selection Bias)	Allocation Concealment (Selection Bias)	Blinding of Partipants and Personnel (Performance Bias)	Blindings of Outcome Assessment (Detection Bias)	Incomplete Outcome Data (Attrition Bias)	Selective Outcome Reporting (Rerpoting Bias)	Other Sources of Bias
De Oliveira et al. [25]	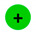	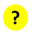	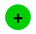	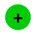	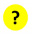	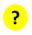	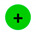
Chou et al. [26]	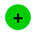	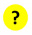	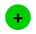	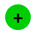	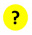	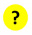	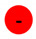
Santos et al. [23]	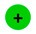	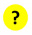	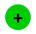	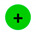	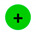	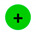	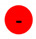
Mohammed et al. [27]	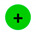	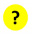	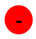	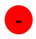	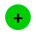	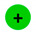	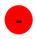
Bohlooli et al. [28]	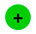	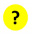	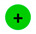	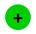	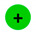	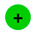	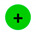
Nakhostin-Roohi et al. [29]	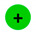	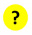	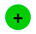	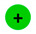	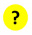	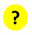	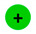
Zoppi et al. [30]	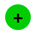	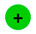	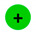	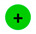	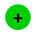	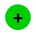	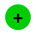
Mastaloudis et al. [31]	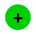	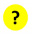	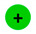	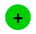	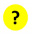	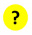	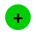
Nie & Lin, [32]	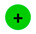	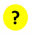	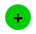	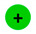	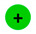	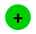	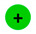
Avery et al. [33]	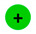	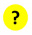	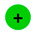	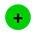	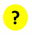	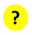	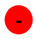
Beaton et al. [34]	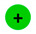	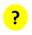	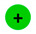	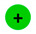	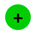	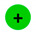	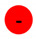
Niess et al. [22]	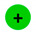	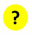	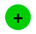	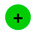	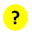	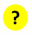	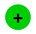
Dawson et al. [20]	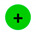	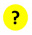	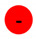	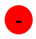	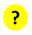	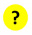	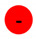
Thompson et al. [24]	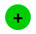	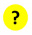	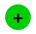	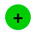	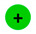	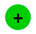	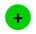
Petersen et al. [35]	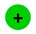	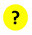	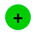	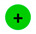	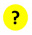	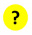	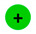
Itoh et al. [36]	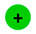	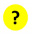	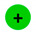	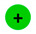	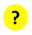	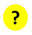	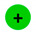
McBride et al. [21]	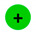	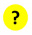	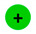	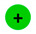	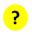	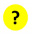	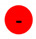
Rokitzki et al. [37]	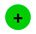	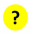	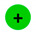	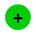	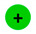	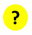	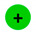
Rokitzi et al. [38]	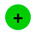	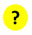	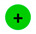	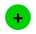	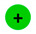	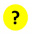	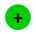
Jakeman & Maxwell, [39]	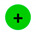	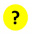	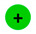	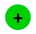	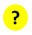	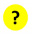	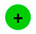
Cannon et al. [40]	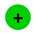	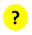	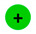	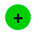	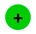	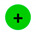	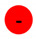

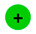
 low risk of bias, 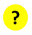 unclear risk of bias 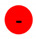 high risk of bias.

**Table 2:** Summary of the studies reviewed examining the effects of an acute dose of VitC or VitE in a single exercise session.

Study	Subjects	Antioxidant Status	Supplementation	Exercise	Blood Samples	Variables other than CK	Results
Santos et al. 2016 [23]	9 physically active men 24.2 (2.17) years	Not reported	VitE (250 mg) 1 h pre-exercise	60 min of exercise (70% VO_2_max) under 3 different conditions (normoxia/hypoxia/hypoxia + SUP in normoxia) as 3 sessions 1 week apart	Before SUP, immediately after, and 1 h after exercise	CK-MB LDH	CK: Levels fell 1 h after exercise in SUP in hypoxia vs. normoxia CK-MB and LDH: Levels fell 1 h after exercise in PLA in normoxia vs. hypoxia
Bohlooli et al. 2012 [28]	16 healthy untrained men SUP (*n* = 8), 21.5 ± 0.8 years PLA (*n* = 8), 22.1 ± 0.68 years	Not reported	VitC (500 mg) 2 h pre-exercise	30 min of treadmill running at 75% VO2max	Before SUP, immediately before and after exercise, and 2 and 24 h after exercise	Plasma VitC MDA	CK: No significant differences between groups Levels elevated immediately post-exercise and 2 h post-exercise in both groups, but only remained elevated 24 h after exercise in PLA MDA: Elevated 2 h post-exercise in PLA vs. SUP but not significantly
Nakhostin-Roohi et al. 2008 [29]	16 healthy untrained males SUP (*n* = 8), 20.9 ± 0.7 years PLA (*n* = 8), 22.1 ± 0.6 years	Not reported	VitC (1000 mg) 2 h pre-exercise	30 min of treadmill running at 75% VO2max	Before SUP, immediately before and after exercise, and 2 and 24 h after exercise	Plasma VitC MDA	CK: No significant differences between groups. Levels elevated immediately post-exercise and 2 h post-exercise in both groups, but only remained elevated 24 h after exercise in PLA MDA: Elevated 2 h post-exercise in PLA vs. SUP but not significantly
Nie and Lin 2004 [32]	16 male junior basketball players SUP (*n* = 8), 16.7 ± 0.3 years PLA (*n* = 8), 16.5 ± 0.2 years	Before the trial: VitC intake (mg/day): SUP: 89 ± 15, PLA: 91 ± 12 During the trial: VitC intake (mg/day): SUP: 87.1 ± 16.6 PLA: 93.6 ± 25.3 VitE intake (mg/day): SUP: 15.1 ± 2.2 PLA: 16.6 ± 3.5	VitC (800 mg) 3 h pre- and 21 h post- exercise	Eccentric exercise trial (10 sets of full-squat jumps at maximum exertion and 30 sets of half-squat jumps)	Before SUP, before exercise, immediately after exercise, and 24 and 48 h post-exercise	Plasma VitC MDA	CK: Elevations reduced at 24 and 48 h post-exercise in SUP vs. PLA MDA: No significant differences between groups
Thompson et al. 2001 [24]	9 healthy physically active men 28.4 ± 1.3 years	During the trial: VitC intake (mg/day): SUP: 120 ± 47 PLA: 119 ± 52 VitE intake (mg/day): SUP: 7 ± 1 PLA: 8 ± 1 Plasma α-toc (µmol/L): SUP: 22.2 ± 2.8 PLA: 21.2 ± 2.9	VitC (1000 mg) 2 h pre-exercise	LIST (two sessions separated by 14 days)	Before SUP, before exercise, immediately after exercise, and 24, 48 and 72 h post-exercise	Plasma VitC MDA	CK: No significant differences between groups Levels above baseline during and after the LIST and peaking 24 h later MDA: Elevated post-exercise in SUP and PLA

Alpha-tocopherol (α-toc), creatine kinase (CK), creatine kinase myocardial band (CK-MB), lactate dehydrogenase (LDH), Loughborough Intermittent Shuttle Test (LIST), maximal oxygen consumption (VO2max), placebo group (PLA), plasma malondialdehyde (MDA), supplemented group (SUP), vitamin C (VitC), and vitamin E (Vit E).

**Table 3:** Summary of studies reviewed examining the effects of chronic supplementation with VitC and/or VitE in one session or several sessions per week of exercise.

Study	Subjects	Antioxidant Status	Supplementation	Exercise	Blood Sample	Variables other than CK	Results
De Oliveira et al. 2019 [25]	21 male football players SUP (*n* = 11) 16.7 ± 0.3 years PLA (*n* = 10) 17.0 ± 0.3 years	Before the trial: VitC intake (mg/day): SUP: 211.4 ± 23 PLA: 151.2 ± 41 VitE intake (mg/day): SUP: 5.1 ± 0.3 PLA: 5.5 ± 0.4	VitC (500 mg/d) and VitE (400 IU of α-toc/d) for 15 d: 7 d before and 7 d after exercise	Exercise-induced oxidative stress protocol: plyometric jumping and strength resistance set to exhaustion	Before exercise and 24, 48 and 72 h post-exercise	Plasma VitC Plasma VitE MDA	CK: No significant differences between groups although levels reduced by 34% in SUP vs. PLA MDA: Levels significantly higher 24 h post-exercise in PLA vs. SUP
Chou et al. 2018 [26]	18 elite male taekwondoists SUP (*n* = 9) 21.0 ± 0.3 years PLA (*n* = 9) 21.3 ± 0.6 years	Not reported	VitC (2000 mg) and VitE (1400 IU/d of dl-α-toc acetate) 3 d before and on the morning of the match	Simulated Olympic-style taekwondo competition (4 matches)	Before the first match, 10 min before each match and 24 h after each match	Mb	CK: No significant differences between group, but significantly lower AUC for SUP vs. PLA Mb: Levels significantly lower 24 h post-exercise and lower AUC in SUP vs. PLA
Mastaloudis et al. 2006 [31]	22 female (*n* = 11) and male (*n* = 11) runners 39 ± 2.5 years	Before the trial: Plasma AA (µmol/L): SUP: 113 ± 14 PLA: 93 ± 11 Plasma α-toc (µmol/L): SUP: 28 ± 2 PLA: 24 ± 2	VitC (1000 mg/d) and VitE (300 IU/d of l-α-tocopheryl acetate) for 6 weeks	50 km ultramarathon trail running	Before SUP, 24 and 1 h before the race in the middle of the race and immediately after, 2 h after and 6 d after the race	LDH Plasma VitC Plasma VitE	CK: No significant differences between groups LDH: No significant differences between groups
Avery et al. 2003 [33]	19 active males SUP (*n* = 9) 22.7 ± 4.1 years PLA (*n* = 9) 22.3 ± 3.6 years	Not reported	VitE (1200 IU/d of RRR-d-α-toc succinate) During 3 weeks before exercise and 10 d after	3 whole-body resistance exercise sessions with 3 days of recovery between sessions.	10 consecutive days in the morning after the initial 21 days of SUP	MDA	CK: No significant differences between groups. AUC significantly higher in SUP vs. PLA MDA: Levels significantly elevated on days 7 and 8 in SUP and PLA
Beaton et al. 2002 [34]	16 sedentary men 20.3 ±1.7 years SUP (*n* = 9) PLA (*n* = 7)	Before the trial: VitE intake (IU/day): SUP and PLA: 16 ± 2	VitE (1200 IU/d of d-α-tocopherol) for 30 d	24 sets of 10 maximal isokinetic eccentric knee extension/flexion contractions	Pre-exercise, 72 h and 7 d post-exercise	Plasma VitE	CK: No significant differences between groups Levels significantly elevated only in PLA 72 h post-exercise. At 7 d post-exercise, levels significantly higher than at baseline in SUP and PLA
Niess et al. 2002 [22]	9 sedentary men 25.3 ± 1.0 years	Before the trial: Plasma AA (µmol/L): 26.6 ± 2.7	VitE (500 IU/d of α-tocopherol) for 8 d	Incremental exercise test and exhaustive continuous run. Two occasions separated with a wash-out of 28 d	1 day before SUP, before exercise and 3, 24 and 48 h after exercise	Plasma VitE Plasma VitC	CK: No significant differences between groups. Levels peaked 24 h post-exercise in both groups, but remained elevated 48 h exercise only in PLA
Dawson et al. 2002 [20]	15 experienced male runners 33 ± 2 years	Before the trial: Plasma AA (µmol/L): SUP: 64.5 ± 4.5 Plasma α-toc (µmol/L): SUP: 8.8 ± 0.8	VitC (500 mg/d) and VitE (500 IU/d) VitC (1000 mg/d) and VitE (1000 IU/d) daily for 4 weeks (4 weeks of washout)	21 km run as fast as possible at baseline an	Before exercise, immediately after exercise and 24 h later	Mb Plasma VitC Plasma Vit E MDA	CK: No significant differences between groups Mb: No significant differences between groups MDA: No significant difference between groups or after exercise compared to baseline
Petersen et al. 2001 [35]	24 male recreational runners SUP (*n* = 12) 28 (23–29) years PLA (*n* = 12) 26 (20-32) years	Not reported	VitC (500 mg/d) and VitE (400 mg/d) for 2 weeks before test and 1 week after test	1.5 h downhill (5%) treadmill run at 75%VO2max	Pre-exercise, immediately post-exercise and 1 h, 2 h, 1 d, 2 d and 7 d later	Plasma VitC Plasma VitE	CK: No significant differences between groups. Levels elevated in both group after exercise and still elevated until day 2 in PLA and until day 7 in SUP
Itoh et al. 2000 [36]	14 physically active males SUP (*n* = 7) 21.7 ± 1.9 years PLA (*n* = 7) 21.1 ± 2.3 years	Not reported	VitE (1200 IU/d of α-toc) for 4 weeks before and during running training (3 weeks)	6 day running training session	Baseline, the day immediately before, the day after, and three weeks after the 6 day running session	LDH Plasma VitE TBA	CK: Levels significantly lower in SUP vs. PLA Elevated in both groups 24 h after exercise and remaining significantly higher 3 weeks later only in PLA. LDH: Levels significantly lower the day after exercise in SUP TBA: Higher in PLA the day before exercise, the day of exercise, the next day and 3 weeks later
McBride et al. 1998 [21]	12 resistance-trained men SUP (*n* = 12) 22.0 ± 0.85 years PLA (*n* = 12) 21.17 ± 0.65 years	Not reported	VitE (1200 IU/d of RRR-d-α-toc succinate) for 2 weeks	Heavy resistance exercise protocol	5 min before exercise, mid-exercise, immediately post-exercise and at 6, 24 and 48 h post-exercise	MDA	CK: Levels significantly lower 24 h after exercise in SUP vs. PLA Significantly elevated after exercise and remaining high 6 and 24 h later in both groups, and only after 48 h in PLA MDA: Significantly elevated immediately post-exercise in SUP and then reduced. Levels increased 6 h post-exercise and remained elevated 24 h post-exercise in PLA
Rokitzki et al. 1994 [37]	16 male runners SUP (*n* = 12) 38.2 ± 7.1 years PLA (*n* = 12) 41.6 ± 9.8 years	Before the trial: Plasma AA (µmol/L): SUP: 50.0 ± 15.3 PLA: 42.6 ± 9.6 Plasma α-toc (µmol/L): SUP: 24.3 ±3.1 PLA: 23.1 ± 6.3	VitC (200 mg/d) and VitE (400 IU/d of α-tocopherol) for 4.5 weeks	Marathon	Baseline, immediately before exercise, immediately after and 24 h later	LDH Plasma VitC Plasma VitE TBARS	CK: Levels significantly lower in SUP vs. PLA 24 h after exercise TBARS: No significant differences between groups. Levels reduced after the race and increased significantly 24 h after the race
Jakeman and Maxwell 1993 [39]	24 physically active males (*n* = 16) and females (*n* = 8) 19.6 years (17.9–21.8)	Before the trial: Plasma AA (µmol/L): 70.5 ± 7.9 Plasma VitE (µmol/L): 26.6 ± 2.7	VitC (400 mg/d) OR VitE (400 IU/d dl-α-toc acetate) for 21 d before and 7 d after exercise	60 min of stepping up and down from a box at a frequency of 24 steps/min	Before SUP, pre- and post-exercise, 60 min after exercise and over 7 days after the exercise	Plasma VitC Plasma VitE	CK: No significant differences between groups
Cannon et al. 1990 [40]	21 sedentary males Group 22–29 years SUP (*n* = 4) PLA (*n* = 5) Group 55–74 years SUP (*n* = 6) PLA (*n* = 6)	Before the trial: Plasma α-toc (µmol/L): Group 22–29 years SUP: 9.05 ± 2.88 PLA: 23.1 ± 6.3 Group 55–74 years SUP: 10.14 ± 2.67 PLA: 10.59 ± 1.48	VitE (800 IU/d of dl-α-tocopherol) for 48 d	Three 15 min periods of downhill running (75% VO2max) on a treadmill	Before exercise, immediately after, and 3 h, 6 h and 1, 2, 5, and 12 days post-exercise	Plasma VitE MDA	CK: Levels significantly higher in older SUP vs. older-PLA before and 2 days post-exercise. Levels rose 24 h after exercise in all the subjects and remained elevated for 2 days but not in older-PLA. Lower CK levels in older-PLA vs. younger-PLA. SUP tended to reduce CK in the younger individuals MDA: No significant changes at any time point in both groups

Alpha-tocopherol (α-toc), area under curve (AUC), ascorbic acid (AA), creatine kinase (CK), lactate dehydrogenase (LDH), maximal oxygen consumption (VO2max), myoglobin (Mb), placebo group (PLA), plasma malondialdehyde (MDA), supplemented group (SUP), thiobarbituric acid (TBA), thiobarbituric acid-reactive substances (TBARS), vitamin C (VitC), and vitamin E (Vit E).

**Table 4:** Summary of studies reviewed examining the effects of chronic supplementation with VitC and/or VitE over a long period of exercise.

Study	Subjects	Antioxidant status	Supplementation	Exercise	Measurements	Variables other than CK	Results
Mohammed et al. 2015 [27]	32 competitive male (*n* = 20) and female (*n* = 12) weightlifters SUP (*n* = 16) 16.5 ± 2.2 years (*n* = 16) 15 ± 1.7 years	Not reported	VitC (500 mg/d) and VitE (400 IU/d of α-toc) for 6 weeks	Routine weightlifting training (2–3 h per day, 5 days per week; 3–8 exercises per session, load 80–100%; 1 to 8 repetitions)	Before SUP and after 6 weeks of intervention	LDH TBARS	CK: No significant differences between groups LDH: No significant differences between groups TBARS: No significant differences between groups
Zoppi et al. 2006 [30]	10 professional male football players SUP (*n* = 5) 18.3 ± 0.5 PLA (*n* = 5) 18 ± 1.0	During the trial: Daily VitC intake (mg) SUP: 28 ± 2.1 PLA: 27 ± 3.5 Daily VitE intake (mg) SUP: 3.4 ± 0.6 PLA: 3.0 ± 0.8	VitC (1000 mg/d) and VitE (800 IU/d of α-toc) for 90 days	Pre-competition season (90 days): stage I (30 days aerobic power), stage II (30 days) strength capacity, and stage III (30 days speed and anaerobic power)	48 h after the last training session in the week before SUP, in the middle and at the end of the season	TBARS	CK: Levels significantly higher at the end of the season in PLA vs. SUP TBARS: Significantly higher at the end of the season in PLA vs. SUP
Rokitzi et al. 1994 [38]	30 professional male cyclists SUP (*n* = 15) 23.4 ± 2.4 years PLA (*n* = 15) 22.5 ± 3.1 years	Before the trial: Plasma α-toc (µmol/L) SUP: 24.9 ± 4.3 PLA: 23.1 ± 8.3	VitE (330 IU/d of dl-α-tocopheryl acetate) For 5 months	Standardized cycle ergometer test (incremental test to exhaustion) before and after 5 months of a mainly aerobic exercise program	Before and after the cycle ergometer test exercise performed before and after the aerobic exercise program	LDH MDA Plasma VitE	CK: Levels significantly lower before and after the cycle ergometer test after 5 months of the aerobic exercise program in SUP vs. PLA MDA: Same pattern as CK LDH: No significant differences between groups

Alpha-tocopherol (α-toc), creatine kinase (CK), lactate dehydrogenase (LDH), placebo group (PLA), plasma malondialdehyde (MDA), supplemented group (SUP), thiobarbituric acid-reactive substances (TBARS), vitamin C (VitC), vitamin E (Vit E)

## Supplementary Materials

The following are available online at https://www.mdpi.com/2076-3921/9/5/372/s1, Table S1: Summary of the studies reviewed examining the effects of an acute dose of VitC or VitE in a single exercise session, Table S2: Summary of studies reviewed examining the effects of chronic supplementation with VitC and/or VitE in one session or several sessions per week of exercise. Table S3: Summary of studies reviewed examining the effects of chronic supplementation with VitC and/or VitE over a long period of exercise.
