# Supplementary material for: Do Antioxidant Vitamins Prevent Exercise-Induced Muscle Damage? A Systematic Review
Source: Antioxidants (Basel). 2020 Apr 29;9(5):372. doi: 10.3390/antiox9050372 (PMC7278664; doi:10.3390/antiox9050372)
Supplement: Supplementary file 1 [file antioxidants-09-00372-s001.pdf]

Supplementary Materials:

Table S1. Summary of the studies reviewed examining the effects of an acute dose of VitC or VitE in a single exercise session.

Study	Subjects	Muscle Function Measurements	Other Variables
Santos et al. 2016 [23]	9 physically active men<br>24.2 (2.17) years	Not reported	Plasma cytokines<br>SaO2%
Bohlooli et al. 2012 [28]	16 healthy untrained men<br>SUP (*n* = 8)<br>21.5 ± 0.8 years<br>PLA (*n* = 8)<br>22.1 ± 0.68 years	Not reported	TAC, total leukocytes, neutrophils, lymphocytes, IL-6, CRP
Nakhostin-Roohi et al. 2008 [29]	16 healthy untrained males<br>SUP (*n* = 8)<br>20.9 ± 0.7 years<br>PLA (*n* = 8)<br>22.1 ± 0.6	Not reported	TAC, leukocytes, IL-6, cortisol
Nie & Lin 2004 [32]	16 male junior basketball players<br>SUP (*n* = 8)<br>16.7 ± 0.3 years<br>PLA (*n* = 8)<br>16.5 ± 0.2 years	Perceived muscle soreness in leg extensors (10 cm visual analogical scale)	Not reported
Thompson et al. 2001 [24]	9 healthy physically active men<br>28.4 ± 1.3 years	General soreness and soreness during hamstrings curl and leg extension (10-point scale)<br>Muscle function in left and right extensors/flexors on an isokinetic dynamometer (% peak torque)<br>RPE	Uric acid, cortisol, total iron<br>AST<br>Blood lactate<br>Heart rate

Aspartate aminotransferase (AST), C-reactive protein (CRP), interleukin-6 (IL-6), percentage oxygen saturation of haemoglobin (SaO2%), placebo group (PLA), rating of perceived exertion (RPE), supplemented group (SUP), total antioxidant capacity (TAC).

Table 2. Summary of studies reviewed examining the effects of chronic supplementation with VitC and/or VitE in one session or several sessions per week of exercise.

Study	Subjects	Muscle Function Measurements	Other Variables
De Oliveira et al. 2019 [25]	21 male football players<br>SUP (n = 11)<br>16.7 ± 0.3 years<br>PLA (n = 10)<br>17.0 ± 0.3 years	Femoral, buttocks, abdomen, total DOMS (10 points visual analogue scale)<br>SPE (10-point scale)<br>Performance parameters: vertical jump, agility, sprint time and fatigue index	GSH/GSSG<br>Plasma FRAP
Chou et al. 2018 [26]	18 elite male taekwondoists<br>SUP (n = 9)<br>21.0 ± 0.3 years<br>PLA (n = 9)<br>21.3 ± 0.6 years	Not reported	Heart rate<br>Lactate<br>Haematocrit and haemolysis<br>Circulating platelet-to-lymphocyte ratio
Mastaloudis et al. 2006 [31]	22 female (n = 11) and male (n = 11) runners<br>39 ± 2.5 years	MVC of the knee extensors and flexors (muscle torque, power deficits, rate of change of muscle torque and power)	CRP
Avery et al. 2003 [33]	19 active males<br>SUP (n = 9)<br>22.7 ± 4.1 years<br>PLA (n = 9)<br>22.3 ± 3.6 years	Perceived muscle soreness in shoulders, chest, quadriceps, hamstring (10 cm visual analogical scale)<br>Performance parameters: maximal strength, explosive power and muscular endurance.	Body composition
Beaton et al. 2002 [34]	16 sedentary men<br>20.3 ± 1.7 years<br>SUP (n = 9)<br>PLA (n = 7)	Concentric peak torque<br>Isometric peak torque<br>Z Band Streaming<br>Perceived muscle soreness (10-point scale)	Presence of macrophages and neutrophils in muscle fibres<br>Edema<br>Antibody staining against desmin and dystrophin
Niess et al. 2002 [22]	9 sedentary men<br>25.3 ± 1.0 years	Maximal blood lactate and heart rates after incremental exercise test	Neutrophils, lymphocytes, monocytes<br>Leukocyte expression of HSP72
Dawson et al. 2002 [20]	15 experienced male runners<br>33 ± 2 years	Not reported	Individual subject ultrastructural muscle changes

Petersen et al. 2001 [35]	24 male recreational runners<br>SUP (*n* = 12)<br>28 (23-29) years<br>PLA (*n* = 12)<br>26 (20–32) years	Not reported	Leukocyte, monocytes
Itoh et al. 2000 [36]	14 physically active males<br>SUP (*n* = 7)<br>21.7 ± 1.9 years<br>PLA (*n* = 7)<br>21.1 ± 2.3 years	Aerobic work capacity (VO2 max and maximal heart rates)	Not reported
McBride et al., 1998 [21]	12 resistance trained men<br>SUP (*n* = 12)<br>22.0 ± 0.85 years<br>PLA (*n* = 12)<br>21.17 ± 0.65 years	RPE<br>Average weight lifted, average percentage decrease in the mean weight lifted, average percentage of the 1RM, average total work (J).	Blood lactate
Rokitzki et al., 1994 [37]	16 male runners<br>SUP (*n* = 12)<br>38.2 ± 7.1 years<br>PLA (*n* = 12)<br>41.6 ± 9.8 years	Not reported	GPT, GOT, GHS-Px, catalase<br>B-Carotene, retinol, uric acid, selenium.<br>bilirubin, haptoglobin, total protein, haematocrit, total protein, erythrocytes
Jakeman & Maxwell 1993 [39]	24 physically active males (*n* = 16) and females (*n* = 8)<br>19.6 years (17.9–21.8)	MVC and ratio of the force generated at 20/50 Hz ratio of triceps surae	Maximal oxygen uptake<br>Lactic acid<br>TAC
Cannon et al. 1990 [40]	21 sedentary males<br>Group 22–29 years<br>PLA (*n* = 5)<br>SUP (*n* = 4)<br>Group 55–74 years<br>PLA (*n* = 6)<br>SUP (*n* = 6)	Not reported	Circulating neutrophils, superoxide release from neutrophils

C-reactive protein (CRP), ferric reducing antioxidant power (FRAP), delayed-onset muscle soreness (DOMS), glutamate oxaloacetate transaminase (GOT), gluthation peroxidase (GSH-Px), glutamate pyruvate transaminase (GPT), maximal O2 uptake (VO2max), maximal voluntary contraction (MVC), one maximal repetition (1RM), oxidized glutathione (GSSG), placebo group (PLA), rating of perceived effort (RPE), reduced glutathione (GSH), subjective perception of effort (SPE), supplemented group (SUP), total antioxidant capacity (TAC).

Table 3. Summary of studies reviewed examining the effects of chronic supplementation with VitC and/or VitE over a long period of exercise.

Study	Subjects	Muscle function measurements	Other variables
Mohammed et al. 2015 [27]	32 competitive male (*n* = 20) and female (*n* = 12) weightlifters<br>SUP (*n* = 16)<br>16.5 ± 2.2 years<br>(*n* = 16)<br>15 ± 1.7 years	Not reported	Anthropometric measurements
Zoppi et al. 2006 [30]	10 professional male football players<br>SUP (*n* = 5)<br>18.3 ± 0.5<br>PLA (*n* = 5)<br>18 ± 1.0	Aerobic capacity (anaerobic threshold)<br>Maximal speed<br>Strength (1RM)	Catalase, glutathione reductase, carbonyl derivatives
Rokitzi et al., 1994 [38]	30 professional male cyclists<br>SUP (*n* = 15)<br>23.4 ± 2.4 years<br>PLA (*n* = 15)<br>22.5 ± 3.1 years	Performance parameters: heart rate, blood lactate concentration and physical performance (W/kg body weight) determined at aerobic threshold and anaerobic threshold	Erythrocytes, haematocrit, haemoglobin, GOT, GPT, hydroxybutyrate-deshydrogenase, alkaline phosphatase, gamma-GT

Gamma glutamyltransferase (gamma-GT), glutamate oxaloacetate transaminase (GOT), glutamate pyruvate transaminase (GPT), one maximal repetition (1RM), placebo group (PLA), supplemented group (SUP).
